# Lack of Recovery From Work and Changes for the Worse in Working Conditions—The Role of Vitality as a Mediator of Lack of Detachment and Sleeping Problems in a 12-Wave Panel Study

**DOI:** 10.1097/JOM.0000000000003434

**Published:** 2025-05-28

**Authors:** Nils D. K. H. Tritschler, Laurenz L. Meier, Achim Elfering

**Affiliations:** From the Institute of Psychology, University Bern, Bern, Bern, Switzerland (N.D.K.H.T., A.E.); Institute of Psychology, University of Neuchâtel, Neuchâtel, Neuchâtel, Switzerland (L.L.M.); and the Swiss National Centre of Competence in Research on Affective Sciences, University of Geneva, Geneva, Geneva, Switzerland (N.D.K.H.T., A.E.).

**Keywords:** job characteristic, reciprocal association, recovery activity, recovery as an outcome, recovery experience, recovery process, reversed effect

## Abstract

**Objective:**

Despite extensive research on occupational stress and impaired recovery, the reverse effects of lack of recovery on work conditions remain largely underexplored.

**Methods:**

Panel questionnaire data from *N* = 4,322 Swiss workers were collected annually across 12 years. Lack of detachment, sleeping problems, vitality, and changes in working conditions were analyzed using growth curve models.

**Results:**

From 2007 to 2019, lack of detachment and sleeping problems increased while vitality and working conditions deteriorated. Vitality predicted the deterioration of working conditions and mediated the detrimental influence of lack of detachment and sleeping problems on working conditions.

**Conclusions:**

Individuals’ recovery status decreased between 2007–2019, while incomplete recovery antecedes a loss of work design quality. Thus, reversed effects of recovery should be a focus of occupational health promotion.

## Abstract 2

This study demonstrates a long-term decline in recovery and working conditions in Swiss workers. Reduced vitality predicted deteriorating working conditions and mediated the influence of detachment and sleeping problems. These findings provide additional reasoning for occupational health interventions and suggest preventive actions by authorities and health specialists against poor recovery.

## Graphical abstract

**Figure:**
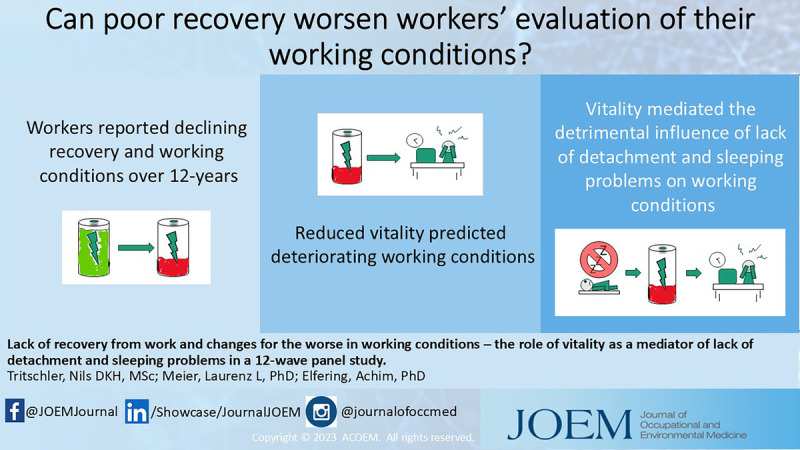

**LEARNING OUTCOMES**

- Describe the role of recovery in shaping working conditions
- Identify the key elements of the scissor model
- Summarize the trends in Swiss workers’ recovery, vitality, and working conditions, emphasizing their interdependence

There is growing evidence that working conditions, recovery from work, and occupational health are reciprocally interdependent.^1–4^ While occupational stress research has traditionally focused on how stressful working conditions impact well-being,^1,5,6^ the reverse effect—how well-being influences working conditions—remains insufficiently understood.^7^ Similarly, although the effects of work conditions on recovery are well documented,^8,9^ research has largely overlooked the potential influence of recovery on working conditions.^10^ Despite several proposed mechanisms explaining reversed effects, no unifying theoretical framework exists.^11^ Furthermore, the few existing studies that examine the effects of recovery on working conditions are constrained by a few measurement occasions,^12,13^ short time frames,^12,14^ and a focus on specific occupational groups, particularly white-collar workers.^15,16^ Additionally, previous research has often adopted a narrow focus, analyzing single indicators of recovery in isolation rather than considering them comprehensively.^14,15^ Addressing these limitations, the current study investigates reverse recovery effects as a critical yet underexplored factor in understanding poor recovery from work and its potential consequences for the working environment. To examine this dynamic, we analyzed data from a large-scale longitudinal study with annual self-reported measurements of multiple recovery indicators and working conditions with a diverse range of occupational groups over a 12-year period.

This study makes three contributions to recovery research. First, guided by the Scissor model,^17^ which postulates the balanced interdependence between recovery and stress states to maintain well-being and performance, we provide novel insight into the relationship between recovery and working conditions. Extending existing research, which mainly focuses on the effect of job characteristics on recovery, this study examines the reverse effects of recovery on the working environment. Second, we advance our understanding of the effect of recovery by differentiating the role of multiple aspects of recovery. Specifically, we examine the effects of recovery experience (lack of detachment), recovery activity (sleep problems), and a recovery outcome (vitality) on working conditions and test how a possible mechanism (vitality as a mediator of the effect of detachment and sleep problems) affects working conditions via increased vitality. Third, by investigating a large 12-year longitudinal dataset, this study provides insight into rarely studied long-term reversed effects.

## THEORETICAL FRAMEWORK AND HYPOTHESES DEVELOPMENT

### The Scissor Model

Recovery is commonly understood as a psychological, physiological, and social process that replenishes personal resources to a baseline level, enabling individuals’ full functional capacity.^18,19^ The scissor model builds on this perspective by emphasizing that from reestablishing resources, the recovery process influences performance and future resilience toward stress, implying two mechanisms on how recovery impacts working conditions:

First, the scissor model underscores recovery’s importance for the resilience of individuals.^17,18^ If the recovery process is disturbed and personal resources cannot be properly replenished, individuals accumulate symptoms of incomplete recovery like a lack of energy and burnout.^20,21^ This state of incomplete recovery diminishes withstanding stressful work: When individuals feel less recovered, the same demands should be appraised as more burdensome because the effort requirements imposed by work are higher relative to the available resources.^20,22,23^ Additionally, since less energy is available to tackle work-related tasks, existing job resources might be perceived as less accessible or helpful since individuals lack the resources to use them properly.^24^ For example, unrecovered individuals perceive customers as more annoying or are too exhausted to apply for adequate work equipment.

Relatedly, because the recovery process reestablishes the functional capacity, it depicts an important preset for performing well. However, if the recovery process is unsuccessful, incomplete, or insufficient, individuals cannot engage effectively with work due to a lack of resources.^20,25^ Because demands are perceived as more burdensome, they must be compensated by increases in effort.^26–28^ However, because of increased effort, work drains more resources, imposing even higher recovery demands to reinstate an equilibrium between current stress states and recovery.^18,21,29^ If this balance is lost, individuals cannot perform well. A diminished performance should result in increases in demands such as time pressure and workload and lost or less accessible job resources like autonomy (ie, individuals need more time for the same tasks, finish fewer tasks, and have less scope of action in fulfilling tasks).

Following this, unlike traditional recovery theories, the Scissor model conceptualizes a potentially increasing gap between reciprocally interdependent stress states and recovery, akin to the opening of scissor blades. Additionally, it encompasses past theoretical assumptions on the underlying mechanisms of reversed effects by emphasizing the timely dynamic of perceptual and behavioral consequences of recovery.

### How Poor Recovery From Work May Worsen Work Conditions

By doing so, the Scissor model addresses the absence of an overarching framework for the various mechanisms proposed for reversed effects.^11^ Traditionally, three mechanisms highlight the detrimental impact of poor recovery on work conditions. First, underlying mechanisms may include a recovery-dependent perception and evaluation of work stressors, called the perceptual hypothesis.^30,31^ Moreover, a restricted recovery status may increase existing and create new work stressors, for example, when people cannot concentrate on work demands because of depleted resources (ie, stressor-creation hypothesis).^32^ In addition, poorly recovered individuals may perform worse and consequently be forced into jobs with higher work stressors and lower job resources.^30,33^ This mechanism called the drift hypothesis, describes how exhausted employees drift to less favorable working environments by getting assigned new but less desirable tasks, transferred to different positions, or laid off.^30^

The Scissor model provides a framework for understanding these mechanisms. As recovery deficits accumulate, individuals perceive work as more demanding and less supportive (perceptual hypothesis). If the equilibrium between stress and recovery is not reinstated, individuals struggle to perform well, which further deteriorates their working conditions (stressor-creation hypothesis). If the imbalance is chronic, an eventual drift into less favorable work environments due to changes in tasks or jobs is implied (drift hypothesis). This interplay illustrates how recovery deficiencies contribute to the long-term deterioration of working conditions based on the interplay between perceptual and behavioral effects.

### Conceptualization of the Recovery Process

Building on the scissor model’s framework, the recovery process can be understood as comprising two interrelated dimensions: recovery activities and recovery experiences, both of which contribute to an individual’s recovered state.^11^ Recovery activities are activities during which recovery occurs,^11^ with sleep being arguably the most important one due to its restorative function, which helps replenish drained resources and contributes to a reduction of fatigue.^34–36^ While recovery activities describe behavioral strategies to restore energy, recovery experiences encompass subjective experiences during leisure time that facilitate unwinding from work.^37^ Detachment constitutes one of the central recovery experiences and refers to individuals’ sense of being mentally distanced from work.^38,39^ Detaching from work promotes mentally recharging resources and depicts a widely recognized indicator for recovery experiences.^38,40^ This study examines sleep problems as a crucial recovery activity and lack of detachment as a key recovery experience involved in the recovery process.

If successful, recovery replenishes personal resources and enhances individuals’ functional capacity (ie, state of readiness for upcoming tasks and well-being^41^). A key indicator of this capacity is vitality, defined as the subjective evaluation of energetic resources.^20,35^ According to the scissor model, sleeping problems, lack of detachment, and low vitality contribute to lower resilience against work-related demands.^16,35,42,43^ Accordingly, energy-depleted individuals will have fewer resources available and perform worse, perceiving a diminished quality in their working conditions due to greater effort requirements while job resources become undervalued, insufficient, or lost. Consequently, the quality of working conditions is expected to deteriorate.

Hypothesis 1: The initial (2007) levels of lack of detachment (a), sleeping problems (b), and vitality (c) predict the development of the quality in changes in the working conditions across 12 years. Specifically, a higher intensity of lack of detachment, more frequent sleeping problems, and less frequent vitality will accelerate the decline in working conditions.

**FIGURE 1:**
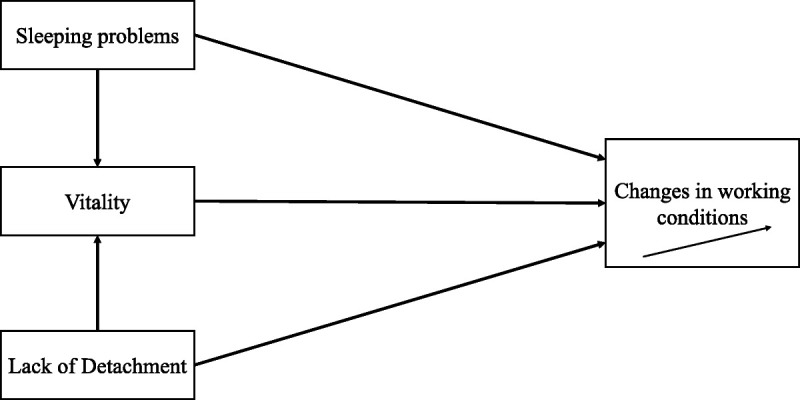
Schematic of study model.

Building on this assumption, it is essential to consider the underlying mechanisms that drive these changes in working conditions. In particular, the role of recovery experiences and activities in shaping vitality provides a theoretical foundation for understanding how initial difficulties in detachment and sleep problems translate into long-term changes in the work conditions. As the Scissor model describes, recovery activities and experiences act as antecedents of functional capacity.^20,44^ Accordingly, individuals who struggle to detach from work and experience more sleep problems are likely to feel less vital. With reduced vitality (ie, lowered functional capacity), job demands tend to be appraised as more burdensome^20,22,23^, and job resources become less accessible.^24^ This suggests that sleep problems and lack of detachment may have an indirect effect on working conditions via lowered vitality (see Fig. 1). Consequently, we presume:

Hypothesis 2: The initial level of vitality mediates the effects of the initial level of (a) lack of detachment and (b) sleeping problems on the slope of the quality of changes in working conditions.

## METHODS

STROBE checklist was utilized to prepare this paper (see SDC 1, http://links.lww.com/JOM/B945). This study is part of the doctoral thesis of the first author.^45^

### Dataset and Study Population

The sample for this research was drawn from the Swiss Household Panel (SHP), a large longitudinal study that started in 1999. Surveys in the SHP are administered annually. Further information can be found in Tillmann and Colleagues^46^ and the supplemental material (see SDC 2, http://links.lww.com/JOM/B946). The analyzed data spans 12 waves from 2007 to 2019, covering the period before the economic crisis in 2008 and ending prior to the COVID-19 pandemic. We selected this timeframe to study a long period of relatively comparable conditions and due to the sample’s evolution. For example, if data from 1999 to 2019 had been included, a large portion of the sample would have been retired by the end of the data collection. Similarly, we tried to avoid potential confounding by the COVID-19 pandemic. Our final dataset included 182′436 observations from *N* = 4,322 individuals. We included data from all working individuals over the age of 15 years who participated in at least six of the measurement occasions. The flowchart of the selection process for this study is available in the supplemental material (see SDC 3, http://links.lww.com/JOM/B947). The final sample consisted of workers from all occupational fields except for forest management, which is considered standard procedure in Switzerland. Women constituted 52% of the sample, with an average education of 13.71 years (SD = 3.13) and average age of 40.22 years (SD = 12.36). Additional demographic information is provided in Table 1.

**TABLE 1:** Detailed Demographic Information for the SHP Sample (*N* = 4′332) Averaged over 12 Years and Rounded

Variable	Answer	*N*	%	Variable	Answer	*N*	%
Sex	Male	2,078	48	Civil status	Single	997	23
	Female	2,254	52		Married	2,343	54.1
Language	French	978	22.6		Separated	60	1.4
	German	2,714	62.7		Divorced	389	9
	Italian	140	3.2		Widowed	61	1.4
Education	Primary school	406	9.4		Registered partnership	16	0.4
	Secondary school	1,984	45.8		Dissolved partnership	1	0
	Tertiary school	1,870	43.2	Night work	Yes	409	9.4
Age group	15–24 years	272	6.3		No	3,002	69.3
	24–44 years	1,588	36.7	Weekend work	Yes	1,553	35.9
	45–64 years	2,221	51.3		No	1,858	42.9
	>65 years	251	5.8	Leadership function	Yes	1,731	40
Sector	Nonpublic services	1,065	24.6		No	1,595	36.8
	Industry & construction	825	19.1	Temporary employment	Yes	223	5.1
	Trade, hospitality, & transportation	893	20.6		No	2,734	63.1
	Public administration, education	724	16.7	Contract type	Full-time	1,728	39.9
	Health & social services	592	13.7		Part-time	1,675	38.7

### Measurements

Variables were assessed using self-reports of single items on Likert scale, repeatedly measured over time for each survey participant.

#### Lack of Detachment

Lack of detachment (ie, lack of “individual’s sense of being away from the work situation,” 38, p. 579) was measured with the item “How difficult do you find it to disconnect from work when the work day is over, if 0 means “not difficult at all” and 10 means “extremely difficult”?”^47^

#### Vitality

Vitality (ie, subjective evaluation of energetic resources) was measured with the item “Are you often plenty of strength, energy and optimism, if 0 means “never” and 10 “always”?”^48^

#### Sleeping Problems

Sleeping problems were measured with the item “During the last 4 weeks, have you suffered from sleeping problems including difficulty in sleeping or insomnia?”^49^ Response options were “not at all” (1), “somewhat” (2), and “very much” (3).

#### Changes in the Working Conditions

Changes in the working conditions were assessed with the item “How have your work conditions evolved since (date of last survey) if 0 means that they have “greatly deteriorated” and 10 “greatly improved”?” (adapted from the study by Diener et al^50^). To simplify interpretation, the item was recoded to (−5) “greatly deteriorated” and (+5) “greatly improved,” with (0) indicating no change.

### Analytic Strategy

#### Reliability Analysis

The reliability of the different single items was estimated by calculating their ICC with a 2-way-mixed-effects-model ANOVA of the type “absolute agreement” and their respective 95% confidence intervals.^51^ Intraclass correlations (ICC) are suitable for concluding single-item retest reliability in repeated assessments.^52,53^ As comprehensively outlined by Koo and Lee,^51^ the ICC, in this sense, refers to the agreement between repeated measurements. The ICC is approximately equivalent to Cronbach’s alpha (for metric items), weighted kappa (for ordinal data), or Maxwells & Piliniers rII (for dichotomous) in the case of repeated administration of the same measurement in large samples.^54–57^ However, as opposed to the other measures, the ICC refers to retest reliability rather than internal consistency.

#### Latent Growth Curve Models

Latent Growth Curve Models (LGM, 57) were analyzed using Mplus 8.^58^ LGMs determine the development of an indicator by regressing several measurement occasions on two latent factors—the intercept and the slope. While the intercept describes the average starting level for the sample, the slope refers to the rate of change per time interval.^59^ LGM can be advanced to measure multiple constructs.^60^ In this study, the effect from the intercept of indicator X on the slope factor Y is of interest, indicating how the initial state of one variable (eg, vitality) is related to the developmental trajectory of another variable (eg, changes in working conditions). Missingness per variable is displayed in Table S1 in the supplemental material (see SDC 4, http://links.lww.com/JOM/B948). Missing data was addressed with FIML (Full-Information-Maximum-Likelihood) to mitigate bias. Confidence intervals for indirect effects were derived with bias-corrected bootstrapping (5000 draws).

#### Univariate Latent Growth Curve Models

In the first step, four univariate LGMs (M1–M4) were estimated for each variable of interest (ie, lack of detachment, vitality, sleeping problems, and changes in working conditions). The latent slope and intercept factor were allowed to covary in each model. The control variables gender, age, and years of education were included in the models as time-invariant covariates.

#### Multivariate Latent Growth Curve Models

To test our hypotheses, we estimated a multivariate LGM (M_5_), including all recovery indicators and changes in working conditions, along with the control variables gender, age, and years of education as time-invariant covariates. The slopes and intercepts of the different recovery indicators and the intercept of working conditions were allowed to covary. In contrast, the slope of working conditions was regressed on the recovery intercepts. Measurement errors were allowed to correlate with measurements within their measurement occasion.

#### Harman’s One-Factor Test

The study addressed common method bias (CMB) introduced by common method variance (CMV) by relying on Harman’s one-factor test.^61^ We used IBM SPSS Statistics (Version 29.0.1.1)^62^ to conduct an EFA (exploratory factor analysis) without rotation in which 19.93% of the variance was explained by a common underlying factor, indicating no biased results.^63,64^

## RESULTS

### Descriptive Analysis

Table 2 presents descriptive statistics such as correlations, means, standard deviations, skewness, and kurtosis of the study variables. Based on the results in Table 2, reliability was sufficient, while kurtosis and skewness indicated non-normal data, but to an extent deemed acceptable for analysis using structural equation modeling (SEM).^66^

**TABLE 2:** Descriptive Statistics and Intercorrelations Between Study Variables (*N* = 4′332)

Variable	M	SD	Skewness	Kurtosis	ICC^a^	ICC 95% CI Lower Bound	ICC 95% CI Upper Bound	Range	1	2	3	4
1. Lack of detachment	3.15	2.19	0.45	−0.69	0.948	0.943	0.952	0–10	x	−0.267***	0.307***	−0.126***
2. Vitality	7.32	1.12	−1.14	2.34	0.909	0.903	0.915	0–10	−0.139***	x	−0.347***	0.232***
3. Sleeping problems	1.37	0.44	1.18	0.28	0.900	0.894	0.906	1–3	0.209***	−0.229***	x	−0.065*
4. Changes in working conditions	0.47	0.58	0.76	2.77	0.618	0.587	0.648	−5–5	0.049	0.099	−0.021	x

Correlations above the diagonal represent between-correlations; within-correlations below the diagonal. Missing data addressed with Full-Information-Maximum-Likelihood.

CI, confidence interval; ICC, intraclass correlation; M, mean; SD = standard deviation.

*P < 0.05; ** *P* < 0.01; *** *P* < 0.001 (two-tailed).

^a^Derived with SPSS and listwise deletion; Interpretation of ICC: <0.5 = poor, ≥0.5 to <0.75 = moderate; ≥75 to< 0.9 = good; >0.9 = excellent.^65^

### Univariate Latent Growth Curve Models

The results for the univariate LGMs are displayed in Table 3 for each variable of interest (ie, lack of detachment, vitality, sleeping problems, and changes in working conditions). Our linear growth curve models reached an acceptable fit.^67^ The significant slope factors indicate a deterioration of the different indicators lack of detachment M_1_(B = 0.023***, SE = 0.004), vitality M_2_(B = −0.021***, SE = 0.002), and sleeping problems M_3_(B = 0.011***, SE = 0.001), as well as a decline in the quality of changes in working conditions M_4_(B = −0.014***, SE = 0.016). For example, the average individual in the sample started at a vitality level of 7.326 in 2007, reporting a significant decline of −0.021 points on the 10-point scale per year, accumulating to −0.252 points over the 12 years of observation.

**TABLE 3:** Results of the Univariate Latent Growth Curve Models (*N* = 4′332)

Model		Lack of Detachment (M_1_)		Vitality (M_2_)		Sleeping Problems (M_3_)		Changes in Working Conditions (M_4_)	
Variable		Estimate	SE	Estimate	SE	Estimate	SE	Estimate	SE
Means:	Intercept	3.241***	0.053	7.391***	0.029	1.313***	0.011	0.487***	0.022
	Slope	0.017**	0.006	−0.027***	0.003	0.008***	0.001	−0.016***	0.003
Covariance:	Slope with intercept	−0.163***	0.010	−0.029***	0.003	−0.006***	0.001	−0.014***	0.003
Variances:	Intercept	4.496***	0.123	1.252***	0.038	0.186***	0.006	0.287***	0.022
	Slope	0.032***	0.001	0.006***	0.000	0.001***	0.000	0.003***	0.000
Model fit:									
χ^2^		638.139		279.14		436.911		265.563	
df		103		103		103		103	
*P*		0.000		0.000		0.000		0.000	
CFI		0.979		0.990		0.980		0.938	
TLI		0.975		0.988		0.977		0.927	
RMSEA		0.035		0.020		0.027		0.019	
RMSEA Lower boundary		0.032		0.017		0.025		0.016	
RMSEA Higher boundary		0.037		0.023		0.030		0.022	

All models adjusted for sex, age, and education in years.

CFI, Comparative Fit Index; df, degrees of freedom; RMSEA, Root Mean Square Error of Approximation; SE, standard error; TLI, Tucker Lewis Index.

*P < 0.05; **P < 0.01; ***P < 0.001 (two-tailed).

### Multivariate Latent Growth Curve Models

To test our hypotheses, we estimated a multivariate LGM (M_5_), including all recovery indicators and changes in working conditions (see methods section for more detail).

#### Direct Effects of Recovery Intercepts on the Slope of Job Characteristics

As reported in Table 4, the resulting model (M_5_) fitted the data well (ie, root mean square error of approximation <0.06, Comparative Fit Index and Tucker Lewis Index > 0.95).^68^ No significant effects were found from the intercepts of lack of detachment and sleeping problems on the slope of changes in the working conditions, discarding hypotheses 1a and 1b. Conversely, and in line with hypothesis 1c, the vitality intercept significantly predicted the slope of the quality of changes in the working conditions (γ_standardized_ = −0.177**, SE = 0.064). Individuals who reported less frequent vitality in 2007 reported a more substantial negative development in the quality of changes in their working conditions till 2018. In contrast, individuals perceiving themselves more frequently as vital in 2007 reported a more stable quality in their working conditions over the 12 years of observation.

**TABLE 4:** Standardized Results of the Multivariate Linear Growth Curve Models (M_5_) (*N* = 4′332)

Variable	Estimate	SE	95% CI_low_	95% CI_high_
Intercept lack of detachment → intercept vitality	−0.181***	0.023	−0.227	−0.138
Intercept lack of detachment → slope changes in working conditions	−0.039	0.057	−0.155	0.069
IE of intercept lack of detachment via vitality	0.030**	0.011	0.010	0.051
Intercept sleeping problems → intercept vitality	−0.288***	0.024	−0.333	−0.239
Intercept sleeping problems → slope changes in working conditions	0.041	0.068	−0.096	0.171
IE of intercept lack of detachment via vitality	0.048**	0.017	0.016	0.084
Intercept vitality → slope changes in working conditions	−0.177**	0.064	−0.309	−0.056
Total indirect effect	0.078**	0.028	0.025	0.134
Model fit				
χ^2^	2704.272			
df	1183			
*P*	0.000			
CFI	0.976			
TLI	0.974			
RMSEA	0.017			
RMSEA Lower boundary	0.016			
RMSEA Higher boundary	0.018			

CI derived with bias-corrected bootstrap (5000 draws).

CFI, Comparative Fit Index; df, degrees of freedom; IE, indirect effect; RMSEA, Root Mean Square Error of Approximation; SE, standard error; TLI, Tucker–Lewis Index; CI derived with bias-corrected bootstrap (5000 draws).

*P < 0.05; **P < 0.01; ***P < 0.001 (two-tailed).

#### Indirect Effects on Working Conditions

To test for the indirect effects on working conditions, we added direct paths from the intercept of sleeping problems and lack of detachment on the intercept of vitality. Table 4 displays the results of this model. The intercept of lack of detachment (γ_standardized_ = −0.181***, SE = 0.023) and sleeping problems (γ_standardized_ = −0.288***, SE = 0.024) predicted the intercept of vitality. Vitality mediated the influence of the intercept of lack of detachment (IE = 0.030**, 95% CI [0.010, 0.051]) and sleeping problems (IE = 0.048**, 95% CI [0.016, 0.084]) on working conditions. The total indirect effect was IE_Total_ = 0.078** (95% CI [0.025, 0.134]). Consistent with hypotheses 2a and 2b, an impaired recovery process (greater difficulties in detaching, higher frequency of sleeping problems) predicted reduced vitality, which in turn predicted a deterioration in the quality of changes in working conditions.

Additional findings on the interrelatedness between the different recovery indicators are reported in the supplemental material (see Table S2, SDC 5, http://links.lww.com/JOM/B949).

## DISCUSSION

This study provides evidence of the potential effects of poor recovery on job characteristics. More specifically, individuals with lower vitality in 2007 showed a more negative development in the changes in their working conditions throughout 12 years. More sleeping problems and higher intensity of lack of detachment from work issues were associated with less frequent feelings of vitality and indirectly increased the deterioration in the quality of working conditions.

Several noteworthy findings emerged from the study. The first finding relates to what element of recovery is associated with the working environment. Opposed to previous evidence, vitality predicted the development of changes in working conditions rather than the two indicators of the recovery process: lack of detachment and sleeping problems. However, in support of the effects of the recovery process,^16,25,42^ we found that recovery activities (ie, sleeping problems) and experiences (ie, lack of detachment) indirectly influenced the quality of changes in the working conditions. Taken together, these results emphasize the complex interplay between the recovery process and the functional capacity of individuals and their impact on the working environment. Furthermore, it underlines prior research, describing recovery activities and experiences as preceding outcomes of recovery, like vitality.^20,44,69,70^

The time-dependency of recovery’s effects on the working environment constitutes another study finding. Past evidence on this matter commonly focuses on relatively short time lags (eg, 1-year, 2-year, or daily/weekly timeframe) by which the long-term time dependency remains largely unknown. We advance this knowledge by presenting evidence that vitality predicted the developmental trajectory of working conditions over 12 years. On the contrary, recovery activities (ie, sleeping problems) and recovery experiences (ie, lack of detachment) did not yield significant direct effects. However, based on prior evidence, we may suspect indicators of the recovery process to exert their influence on the working environment over shorter time periods (daily-diary – 1–2 years lags).^14,16,42^

Moreover, it seems important to note that even small effects, as found for the mediations in this study, may contribute to meaningful changes in the working environment over time. For example, contrastingly to a one-time effect, if the indirect effect of lack of detachment on working conditions is analyzed over 12 years, this translates to 12 times this effect due to accumulation. Notably, this assumption also holds for the here-found direct effect. As such, poor recovery results in a more profound decline in the quality of job characteristics, as one might expect. Future studies may investigate whether these effects accelerate for certain groups of individuals or stay relatively stable over time.

### Theoretical Implications

**FIGURE 2:**
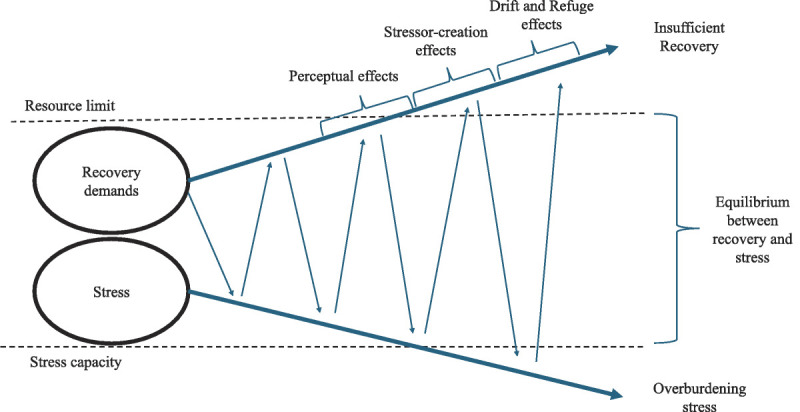
Scissor Model with reversed effects. Adapted by permission from K.W. Kallus and M. Kellmann, “Burnout in Athletes and Coaches,” in Emotions in Sport, edited by Y. Hanin (Human Kinetics, 2000) 209–230.

The time dependency and stability of reversed effects depict two crucial aspects for adapting a theory that accounts for the dynamic nature of the recovery process and its influence on working conditions. We propose the Scissor model as an overarching theory, emphasizing recovery’s role for the resilience and the functional capacity of individuals while highlighting the reciprocal interplay between stress states and recovery demands. As such, the scissor model underscores the influence of the current capacity on the appraisal of the environment. For example, a professional athlete will appraise a 100-meter sprint as more burdensome if she must complete the sprint immediately after a marathon due to the increased fatigue and lack of energy imposed. In our opinion, these preliminary effects suggested by the scissor model are mostly comparable to the assumptions based on the perceptual hypothesis that refers to changes in the perception of demands and resources depending on the health status. However, the findings of the study raise an interesting question—if such perceptual effects exist, is this influence solely limited to the perception of demands and resources (ie, subjective change), or does this spill over to objective impairments of job characteristics as mentioned within the stressor-creation^32^ and the drift hypothesis.^18,33^ For example, the scissor model implies that decrements in the functional capacity led to more unfinished tasks, resulting in objectively more job demands (eg, increased workload and time pressure) and a loss of resources such as autonomy (ie, stressor-creation hypothesis).^71^ Furthermore, if the balance between recovery demands and stress states cannot be reinstated, symptoms of incomplete recovery accumulate and individuals potentially drift to more unfavorable working conditions (ie, drift hypothesis^18^). From the athlete’s lens, if the same individual is confronted with repeated marathons followed by 100-meter sprints, an accumulation of fatigue and a lower functional capacity are likely. Consequently, the athlete will perform worse, which may create conflicts with supervisors or teammates (stressor-creation hypothesis). In the most extreme case—ie, if the recovery process is continuously unsuccessful, disturbed, or incomplete—the scissor model describes how recovery demands (increasing) and stress capacity (decreasing) increasingly drift apart (see Fig. 2). In this case, the athlete exceeds her resource limit and is unable to reestablish her functional capacity. As a result, supervisors may ultimately assign new tasks, positions, or jobs (drift hypothesis).

Another interesting avenue to follow is considering the type of appraisal of demands and how this relates to recovery. Previous studies found that job demands’ effects on recovery vary based on their appraisal.^69,72^ This has not been investigated for reversed effects, while differential effects may occur (see the study by Casper et al^73^ for notable exception). For example, challenge demands might be impaired while hindrance demands are accelerated by poor recovery. In other words, if individuals lack energy, they are less likely to appraise demands as challenged due to insufficient resources, perceiving them as more hindering.

### Practical Implications

The study yields several implications. First, a deterioration in the recovery processes and the vitality was visible in our sample. These findings align with prior studies on increasingly strained workers in Switzerland.^74–77^ The unfavorable development might be due to various reasons, such as constant availability during non-work hours or other changes due to digitalization.^39^ However, it is crucial to note that, on average, changes in the working conditions still indicated an improvement in job characteristics for workers, as evident from their value above 0 – regardless of their increasingly adverse development (ie, negative slopes).

Secondly, our analysis highlights the importance of recovery for the working environment. In the face of prior studies that mainly investigated the effects of job characteristics on recovery, our findings indicate a bidirectional relationship. Taken together, the study should encourage occupational health specialists to identify causes for the deterioration of employees’ recovery. Additionally, they should focus on improving recovery to avoid a potentially detrimental cycle where high job demands and low resources lead to poor recovery, which, in turn, worsens job characteristics even further.

### Limitations

There are several limitations of this study. First, the study relied on a WEIRD sample (western, educated, industrialized, rich, and democratic), limiting generalizations to other populations. Additionally, the sample is not representative of the Swiss working population. For example, immigrant workers, depicting 32.4% of the workforce, are lacking (vs. ~10% in our sample).^78^ The small number of 10% in this sample may signal that our research might underestimate the development of working conditions in the Swiss working population because foreign workers are more likely employed in precarious working arrangements and typically show poorer health than Swiss workers.^79,80^ However, the sample aligns with previous studies on the working population in Switzerland since immigrant workers are typically not sufficiently included in such relatively representative studies.^81,82^

Furthermore, the selected timeframe between 2007 and 2019 depicts another limitation. Although the selected timeframe helps to discern potential confounding, the COVID-19 pandemic might yield additional insights into reversed effects. For example, the increased number of employees forced to work from home by the COVID-19 pandemic introduced new demands and additional challenges to mental health due to diminished boundaries between work and private life, resulting in conflicts between both domains and greater difficulties in detaching.^83–85^ As such, an acceleration of reversed effects might have occurred. On the other hand, the pandemic likely led to a longer-lasting transition to remote work, which may hold some positive effects on the health of employees and could counteract the development of deteriorating health in the working population to some degree.^85,86^ However, it seems important to note that the positive development is likely limited to certain groups of workers.^87^

Third, the results of LGMs do not permit causal interpretation of effects, regardless of the longitudinal nature of data. Therefore, our analysis method does not allow us to draw causal inferences.

Another limitation of this study stems from the use of single items. Single items have practical advantages over scales with multiple items in extended interviews or surveys^88,89^ but are criticized for having less psychometric quality.^90,91^ Nevertheless, some studies found single items for constructs like fatigue and job satisfaction to be reliable and of acceptable validity.^89,92–94^ Regarding our analysis, we can conclude that our single items are of sufficient reliability. However, one of the major arguments against single items concerning their limited predictive validity should increase the chances of missing out on effects rather than falsely finding them. In the same instance, acknowledging impairments using single items, like the impossibility of evaluating measurement invariance of indicators, should be considered. Frequently overlooked in research, measurement invariance depicts a mandatory condition to ensure proper interpretation of the results.^95,96^ In sum, concerns about the validity of our measures must be accepted, and future studies should rely on multiple items of high psychometric quality to ensure results of higher validity.

Self-reported measures potentially promote CMB introduced by CMV.^97^ While some conditions of this research are beneficial for CMB, like the use of single items or the simultaneous collection of criterion and predictor,^63,98^ others reduce it, such as appropriate scaling to reduce anchor effects,^64^ longitudinal analysis methods^98^ or analysis on the within-level.^99^ The study addressed CMV by relying on Harman’s One-factor test,^61^ which showed no evidence of bias (see methods). Considering everything, severely biased results are unlikely, although it should be noted that estimation with different methods is typically advised.^53,100^

## CONCLUSIONS

To conclude, this study demonstrates the effect of recovery outcomes (ie, vitality) on the quality of working conditions. Additionally, this study underlines how different aspects of the recovery process indirectly influence the working environment. Our findings suggest that the poor recovery of employees in 2007 predicted a more substantial negative development of job characteristics over 12 years. Consequently, this research emphasizes the importance of recovery for the development of demands and resources at work and warrants considering the implication of recovery for reasons beyond its value for workers’ health. As such, this study contributes additional reasoning for occupational health interventions and preventive actions by authorities and health specialists against poor recovery. Future research should explore boundary conditions of the different underlying mechanisms (perceptual, stressor-creation, and drift hypothesis), effects on differently appraised demands, and the potential acceleration of effects to gain a better understanding of how recovery contributes to the development of the working environments of individuals.
